# Atrial electrogram amplitude–guided Bachmann bundle pacing implantation: A single-center experience

**DOI:** 10.1016/j.hroo.2026.06.003

**Published:** 2026-06-12

**Authors:** Daisuke Yoshimoto, Yuichiro Sakamoto, Yuko Uemura, Ryo Yamaguchi, Hirokazu Naganawa, Takahiko Suzuki

**Affiliations:** Toyohashi Heart Center, Aichi, Japan

**Keywords:** Bachmann bundle pacing, Delivery catheter, Interatrial conduction delay, Lumenless pacing lead, Right atrial septal pacing

## Abstract

**Background:**

Bachmann bundle pacing (BBp) seems to be associated with reduced incidence and persistence of atrial fibrillation. The Bachmann bundle area is located at the junction between the superior vena cava and the right atrium. Pacemaker leads were implanted just inferior to a site demonstrating an abrupt increase in electrogram amplitude as the lead delivery system was advanced inferoseptally from the superior vena cava toward the right atrium as long as adequate sensing and capture thresholds were demonstrated. Subsequent to the implant, paced P waves were evaluated for evidence of Bachmann bundle capture.

**Objective:**

This study aimed to evaluate the feasibility, safety, and short-term outcomes of an atrial electrogram amplitude–guided implantation strategy for BBp.

**Methods:**

We retrospectively included 50 patients who underwent dual-chamber pacemaker or implantable cardioverter-defibrillator implantation with attempted BBp between September 2023 and December 2024. The atrial pacing lead was fixed just below the site demonstrating an abrupt change in atrial electrogram amplitude, typically exceeding 1 mV.

**Results:**

Atrial septal lead implantation was achieved in all patients, with successful BBp (meeting electrocardiogram criteria) in 44 (88%). The median mapping time was 134.5 seconds. Immediately after fixing the lead, the mean capture threshold was 1.86 ± 1.00 V at 0.4 ms, improving to 0.834 ± 0.315 V by the end of the procedure. During the 6-month follow-up, pacing parameters remained stable, with no lead dislodgement, pericardial effusion, or device-related infection.

**Conclusion:**

BBp using the lumenless pacing lead and delivery catheter, guided by an atrial electrogram amplitude–based strategy, is safe, feasible, and reproducible.

## Graphical abstract

**Figure:**
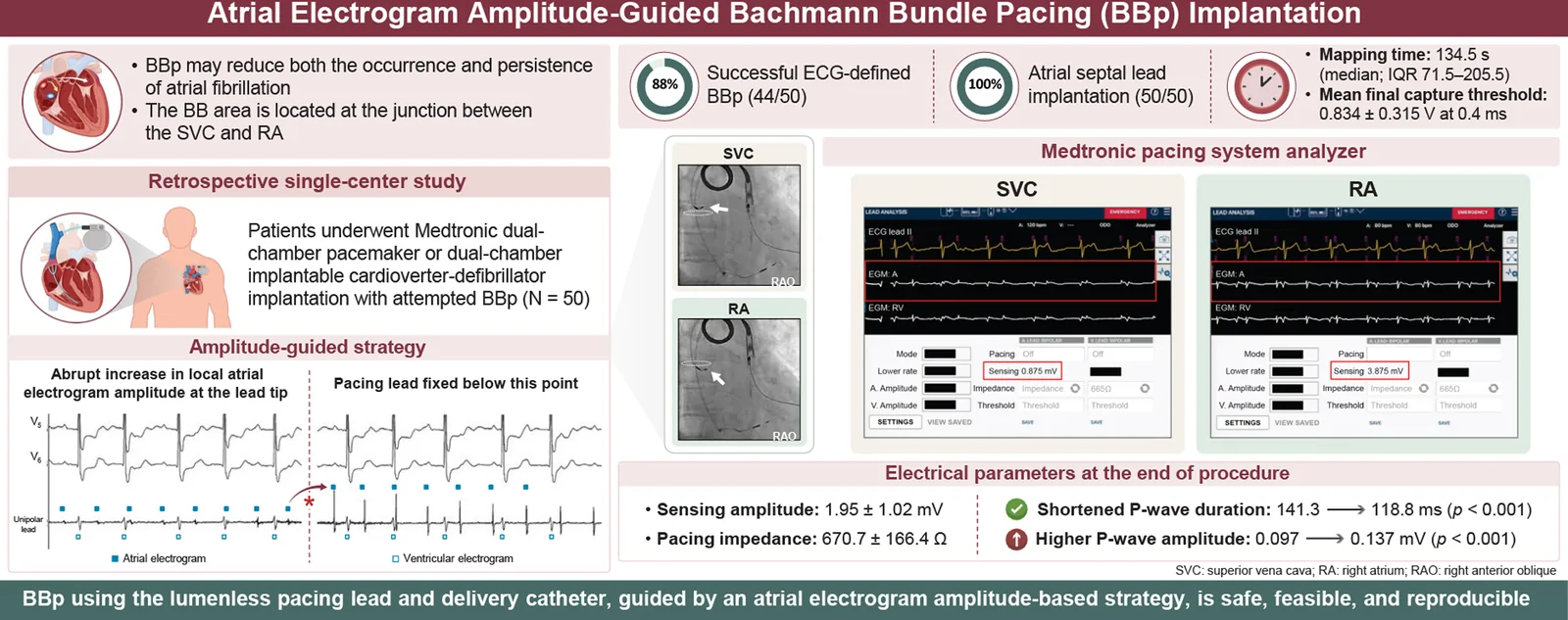

**Key Findings**

▪ An atrial electrogram amplitude–guided approach enables reproducible identification of the superior vena cava–right atrial junction and facilitates reliable high atrial septal lead implantation fulfilling electrocardiogram-defined Bachmann bundle pacing (BBp) criteria.
▪ Transient elevation of pacing thresholds after atrial septal lead fixation frequently improves within several minutes, suggesting that sufficient recovery time should be allowed before lead repositioning.
▪ Amplitude-guided implantation represents a practical strategy for BBp that does not require specialized electrophysiology recording systems and may therefore be broadly applicable in routine device implantation.

## Introduction

Traditionally, the right atrial appendage (RAA) has been the most common site for atrial lead implantation. However, the P-wave duration (PWD) during RAA pacing (RAAp) is broader than in sinus rhythm, leading to interatrial conduction delay and increasing the risk of atrial fibrillation (AF) development.^1^^,^^2^ In contrast, right atrial septal pacing (RASp) has the potential to achieve better biatrial synchrony than RAAp, and its clinical efficacy has been anticipated. In several prospective randomized studies comparing RAAp and RASp, the latter was shown to significantly reduce AF burden. Subsequently, meta-analysis of 12 randomized trials demonstrated that RASp had safety and tolerability comparable with RAAp and was associated with a reduced AF burden; however, no significant differences were observed between the 2 groups in terms of AF persistence or recurrence.^3^

In 2022, Infeld et al^4^ reported that Bachmann bundle pacing (BBp), characterized by a P-wave morphology similar to sinus rhythm and a shortened PWD, significantly suppressed the incidence and persistence of AF compared with RAAp and nonspecific atrial septal pacing, which was RASp without BBp. Histologically, the Bachmann bundle (BB) has distinctive characteristics, and studies in canines have demonstrated that the conduction velocity of the BB is several times faster than that of the surrounding atrial myocardium.^5^^,^^6^ Furthermore, Anderson et al^7^ used the histologic sections of both human and swine hearts to demonstrate that the BB consists of the parallel alignment of atrial cardiomyocytes aggregated together, connecting the right and left atria, which can function as a substrate for rapid interatrial conduction. They further suggested that selective pacing within this region may currently represent a substantially more favorable option than traditional RAAp. Therefore, lead implantation at the high atrial septum that meets electrocardiogram (ECG) criteria, including shortened PWD and increased P-wave amplitude, seems critical for achieving clinical efficacy across various RASp sites. These findings have increased interest in BBp as a physiological atrial pacing method.

Nevertheless, the RAA remains the most common site for atrial lead implantation among operators. This may be attributable to the fact that most pacing leads are stylet driven, and standard stylets for atrial lead implantation are fundamentally designed for RAA placement, making it a familiar and conventional approach. Although the implantation technique for BBp using a stylet-driven lead has been described,^8^ no dedicated stylet is currently available. Therefore, this technique requires customized stylet shaping and specific manipulation skills, making the procedure highly dependent on operator experience.

In contrast, a fixed curve delivery catheter that has 3-dimensional (3D) curves reliably engages the atrial septum and allows a lumenless pacing lead to be easily and reproducibly implanted at the atrial septum. For lead implantation at the high right atrial (RA) septum that meets the BBp ECG criteria associated with clinical benefits, several procedural strategies have been reported to assess the optimal implantation site and achieve successful BBp.^9^^,^^10^ These include evaluation of the paced P-wave morphology, assessment of fragmented late potentials considered to represent BB potential on unipolar recordings at the lead tip, and contrast injection from the tip of the Medtronic delivery catheter. However, particularly in institutions where a recording system for evaluating lead-tip electrograms, P-wave morphology, or PWD during implantation is not routinely available, practical and reproducible approaches for achieving BBp in clinical practice remain limited.

**Figure 1:**
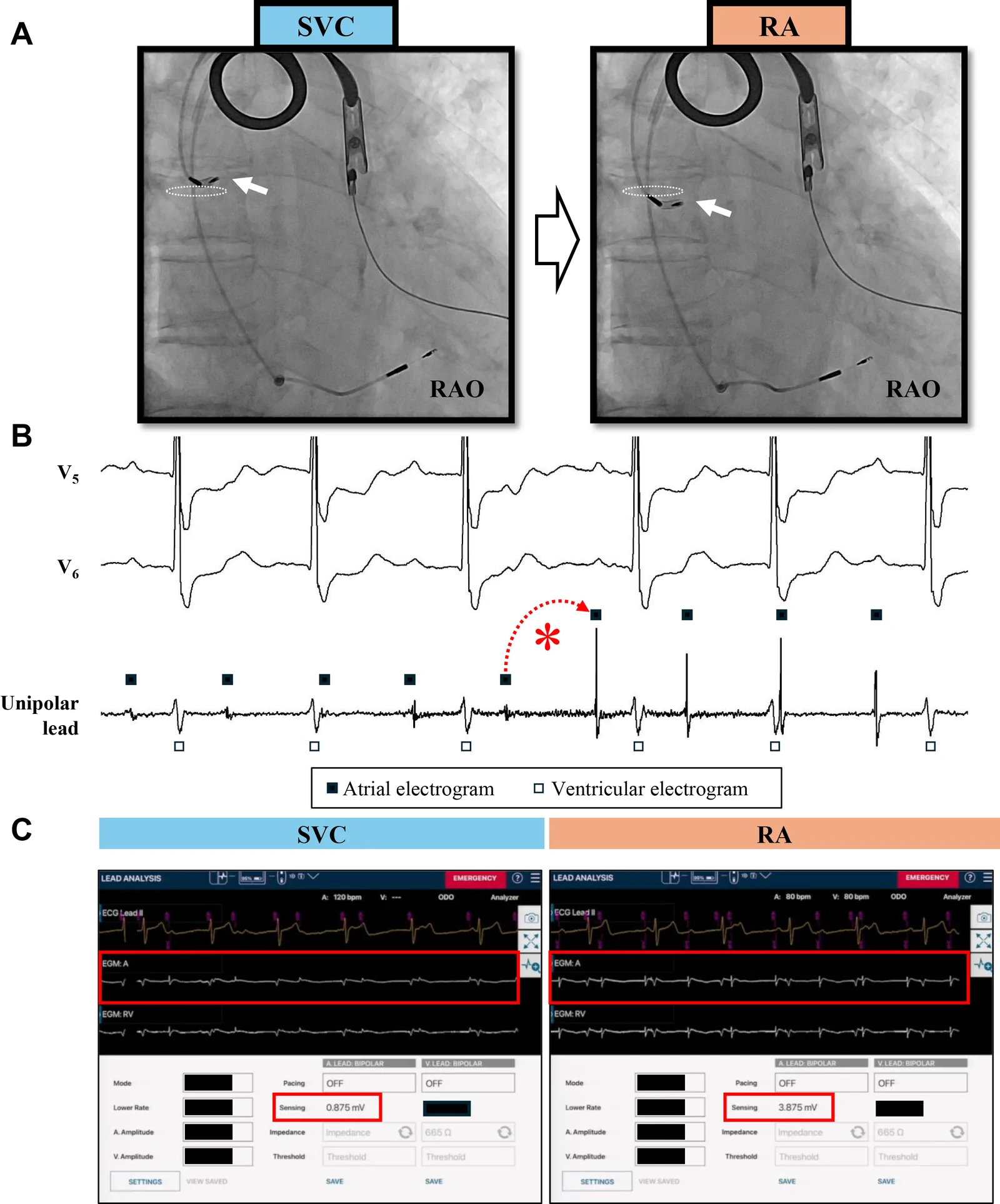
Atrial lead implantation using an atrial electrogram amplitude–guided approach. **A:** Fluoroscopic images of unipolar lead mapping (*arrows*) performed using a C315S5 delivery catheter advanced downward along the septal aspect from the SVC to identify the SVC–RA junction (*ellipse with dotted line*) by detecting the abrupt change in local atrial electrogram amplitude. **B:** Representative electrogram tracing obtained from the electrophysiology recording system during unipolar mapping using the pacing lead. Near-field atrial and far-field ventricular electrograms are simultaneously recorded. The SVC–RA junction is identified by an abrupt increase in the unipolar atrial electrogram amplitude (*asterisk*). **C:** Representative screenshot from the Medtronic pacing system analyzer. During the procedure, atrial electrogram amplitudes measured by the pacing system analyzer are used to identify the SVC–RA junction, defined by an abrupt change in the unipolar atrial electrogram amplitude, and the lead is fixed just below this point. RA = right atrium; RAO = right anterior oblique; SVC = superior vena cava.

The BB area is located at the junction between the superior vena cava (SVC) and the RA. Therefore, in our implantation strategy, pacemaker leads were implanted just below a site demonstrating an abrupt increase in local atrial electrogram amplitude recorded in unipolar configuration at the lead tip (Figure 1). Using this amplitude-guided atrial lead implantation approach, we evaluated the success rate of BBp implantation, pacing parameters and paced P-wave characteristics at implantation, procedure-related complications, and short-term clinical outcomes.

To the best of our knowledge, this is the first study to systematically evaluate an atrial electrogram amplitude–guided approach for BBp using a delivery catheter system.

## Methods

### Patient selection

Following the current Japanese Cardiology Society/Japanese Heart Rhythm Society guidelines,^11^ all patients who underwent Medtronic dual-chamber pacemaker or dual-chamber implantable cardioverter-defibrillator (ICD) implantation, whose atrial leads were implanted using a C315S5 delivery catheter (Medtronic Inc, Minneapolis, MN) and a lumenless pacing lead (model 3830 SelectSecure; Medtronic Inc) to attempt lead implantation in the BB area between September 2023 and December 2024, were included in our institution’s observational, retrospective study. All patients provided an informed consent, and the ethics review board approved this study.

### Procedure

After the ventricular lead implantation, atrial lead implantation was performed using a Medtronic C315S5 delivery catheter and a SelectSecure pacing lead. All procedures were performed by 1 of 4 operators experienced in conduction system pacing (D.Y., Y.U., R.Y., or H.N.).

#### Atrial lead implantation using an atrial electrogram amplitude–guided approach for BBp

A SelectSecure pacing lead with a C315S5 delivery catheter was used in all patients. The delivery catheter was advanced into the RA using a guidewire through the left or right subclavian vein, and the pacing lead was advanced through the catheter into the RA septum. A unipolar atrial electrogram at the lead tip was measured using a Medtronic pacing system analyzer (PSA), and the electrograms were simultaneously recorded on an electrophysiology (EP) recording system (Bard Electrophysiology Lab System, Lowell, MA) with a 30–500 Hz filter via a jumper cable. Intraprocedural electrical parameters were acquired and measured using the PSA, whereas the EP recording system was used exclusively for recording purposes for retrospective analysis. Starting from a location where low-amplitude atrial electrograms are recorded on the atrial septum, local atrial electrogram amplitudes were measured during sinus rhythm.

In this setting, the 3D shape of the delivery catheter is designed such that the catheter tip naturally directs toward the atrial septum. At sufficiently high positions, the atrial transverse diameter is relatively narrow, allowing the catheter tip to maintain consistent contact with the septal wall. The catheter has a soft tip, and by positioning the lead screw flush with the distal end of the catheter during manipulation, unipolar electrograms can be recorded with the lead in this retracted position, and mapping can be performed safely without increasing the risk of venous dissection or myocardial injury. For accurate measurement of electrogram amplitude, firm contact between the lead and the septal wall is required. Therefore, within the region of interest, the catheter was kept stationary while the lead was advanced only minimally beyond the catheter tip. Atrial electrogram amplitudes were measured while confirming slight backward displacement of the catheter owing to counterforce, indicating adequate septal contact. If the lead was observed to slide along the septal wall, the lead was first retracted into the catheter, and counterclockwise torque was applied to the catheter, allowing the catheter tip to re-engage the septum and facilitate proper contact of the lead with the septal wall.

Mapping was then performed downward along the septal aspect from the low-voltage region within the SVC, where the signal amplitude fell below the sensitivity threshold at 0.3 mV. The site at which the atrial electrogram amplitude demonstrated an abrupt increase, typically exceeding 1 mV, and the electrogram morphology changed from a relatively dull to a sharp potential was identified as the SVC–RA junction. During mapping, unipolar electrogram amplitudes were measured by an engineer using a Medtronic PSA and verbally reported to the operator, who determined the location of the SVC–RA junction. However, because this study was retrospective, the electrogram amplitudes used for analysis were not those measured by the PSA during the procedure. Instead, all amplitudes were retrospectively quantified from continuously recorded electrograms obtained in the EP recording system.

A few millimeters below this site demonstrating an abrupt increase in electrogram amplitude, the lead was fixed into position after confirming consistent atrial capture at 2.0 V at 0.4 ms. At that time, a detailed assessment, such as the capture threshold and paced P-wave morphology, was not performed. During mapping before lead fixation, if the atrial capture threshold near the site of an abrupt increase in the amplitude was >2.0 V at 0.4 ms, the mapping was sequentially extended downward along the atrial septum, and the lead was fixed at the site where atrial capture was achieved at ≤2.0 V at 0.4 ms. In cases where the entire atrial septum exhibited low voltage and a sudden increase in atrial electrogram amplitude could not detected, the atrial septum was mapped using the capture threshold as a reference, and the lead was fixed at the site where an acceptable capture threshold was observed.

After fixing the lead, the catheter was withdrawn. Because the atrial transverse diameter is narrow at the level of the SVC–RA junction, the catheter was pulled back to create slack in the lead, as previously described, which involved advancing the lead and catheter together into the RA before withdrawing the catheter.^10^

Subsequently, the bipolar atrial electrogram amplitude, lead impedance, and atrial capture threshold were measured. Paced P-wave morphology was not considered during operation. After connecting the lead to the generator, a 12-lead ECG was evaluated using the EP recording system to determine whether it met the BBp ECG criteria previously reported:^4^

1) Vector similar to normal sinus rhythm, with upright P waves in leads I, II, III, and aVF and a biphasic or negative P wave in lead V1
2) Peak and symmetric P waves in the inferior limb lead, with a paced P-wave amplitude typically greater than the sinus P-wave amplitude
3) Narrowed PWD compared with sinus PWD, typically >10 ms if baseline interatrial conduction delay was present

Among patients who did not meet the BBp ECG criteria, atrial septal pacing was classified by fluoroscopy, distinguishing between high- and low-septal pacing.

### Device programs and follow-up

At implantation, the device was programmed with a lower rate limit of 60 beats/min and an upper tracking rate of 130 beats/min. In patients requiring ventricular pacing, the pacing mode was set to DDD, whereas the managed ventricular pacing function was activated in those not expected to require ventricular pacing. Although the rate response function was nominally turned off, it was subsequently activated at the discretion of the attending physician in patients with a high percentage of atrial pacing. The atrial antitachycardia pacing function was activated in all patients. Device parameters and AF/atrial tachyarrhythmia episodes were evaluated approximately 1 week after implantation during hospitalization and at approximately 1 and 6 months during outpatient follow-up. The diagnosis of AF/atrial tachyarrhythmia was based on device-registered electrograms and was defined as previously reported.^12^

### Statistical analysis

Continuous variables are presented as means ± standard deviations or medians with interquartile ranges (IQRs) and compared using the Student *t* test or Mann–Whitney U test. Categorical variables were compared using the χ^2^ test. Statistical significance was defined as a 2-tailed *P* < .05. All statistical analyses were performed using IBM SPSS Statistics version 22 (IBM Corp, Armonk, NY).

## Results

65 consecutive patients undergoing attempted lead implantation in the BB area using the delivery catheter and the lumenless pacing lead were enrolled. Of these, 15 patients were excluded because of inaccurate evaluation of intraoperative ECG or local electrograms of lead tip caused by noise or insufficient temporal recordings of pacing parameters from the EP recording system. The remaining 50 patients (mean age 78.0 ± 9.9 years; 76.0% male) were retrospectively analyzed (Table 1). All patients underwent device interrogation and follow-up at the outpatient clinic. Of the implanted devices, 94.0% were dual-chamber pacemakers, and 6.0% were dual-chamber ICDs. The indications for dual-chamber pacemaker implantation included sinus node dysfunction in 10.0% and atrioventricular block in 84.0%. All ICD implantations were performed for secondary prevention of ventricular arrhythmias. A history of AF was noted in 16.0% of patients, and all patients had sinus rhythm at the time of the procedure. Preoperative transthoracic echocardiography revealed preserved cardiac function in most patients, with a mean left ventricular ejection fraction of 60.5% ± 8.2%. Furthermore, mildly reduced systolic function, with a left ventricular ejection fraction between 40% and 50%, was observed in 14.0% of patients.

**Table 1:** Baseline patient characteristics (n = 50)

Characteristic	
Age, y	78.0 ± 9.9
Sex, male	28 (56.0)
Body mass index, kg/m^2^	22.4 ± 4.0
Medical history	
Hypertension	27 (54.0)
Diabetes mellitus	17 (34.0)
Coronary artery disease	9 (18.0)
Previous stroke	2 (4.0)
Atrial fibrillation	8 (16.0)
Echocardiography	
LVEF, %	60.5 ± 8.2
Left atrial diameter, mm	37.5 ± 6.2
Device type	
Pacemaker	47 (94.0)
Sinus node dysfunction	5 (10.0)
Atrioventricular block	42 (84.0)
ICD	3 (6.0)
Cardiac sarcoidosis	1 (2.0)
Dilated cardiomyopathy	1 (2.0)
ARVC	1 (2.0)

Values are given as numbers (%) or means ± standard deviations.

ARVC = arrhythmogenic right ventricular cardiomyopathy; ICD = implantable cardioverter-defibrillator; LVEF = left ventricular ejection fraction.

### Procedural outcomes, lead parameters, and ECG characteristics

**Figure 2:**
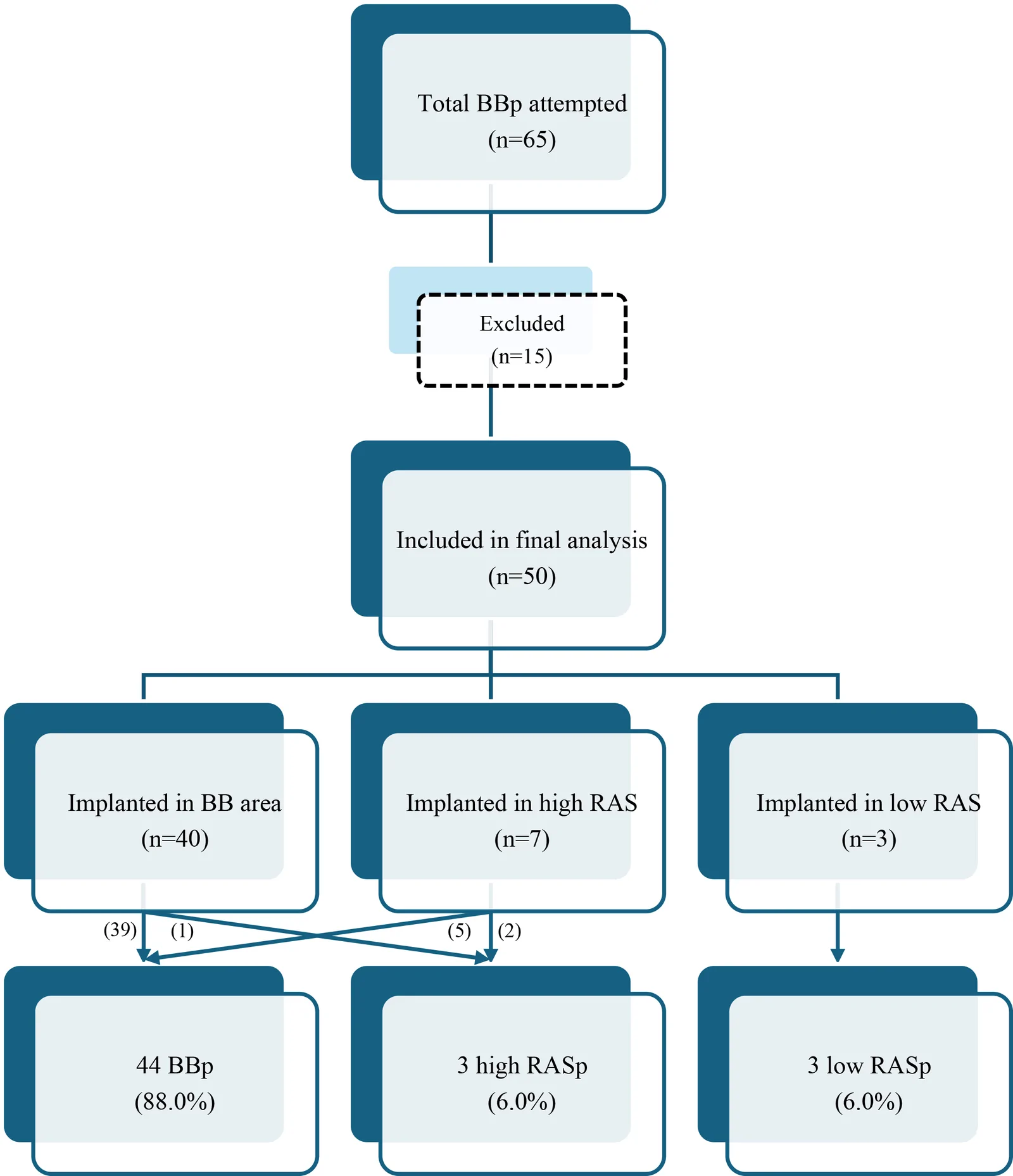
Schematic representation of the study. BB = Bachmann bundle; BBp = Bachmann bundle pacing; RAS = right atrial septum; RASp = right atrial septal pacing.

**Figure 3:**
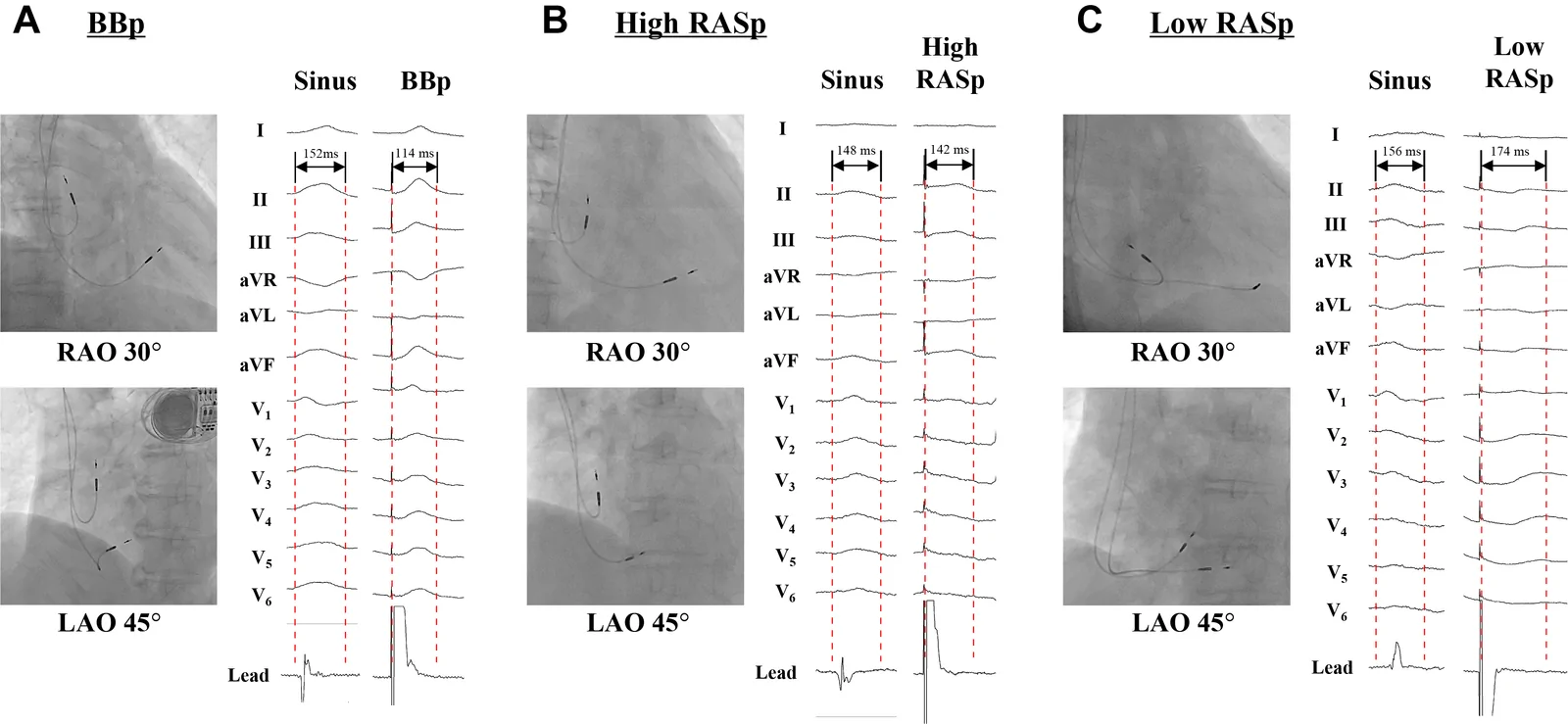
Atrial lead fluoroscopy in RAO 30° and LAO 45° projection, and P-wave morphology at 100 mm/s in each atrial pacing site. **A:** Fluoroscopic images of Bachmann bundle lead position and the PWD during sinus rhythm and BBp are shown. **B:** Fluoroscopic images of high atrial septum lead position and the PWD during sinus rhythm and high RASp are shown. The paced P wave is not symmetrical in the inferior limb leads, and although interatrial conduction delay is present (sinus PWD >140 ms), the paced PWD is not shortened by more than 10 ms, thereby failing to meet BBp ECG criteria 2 and 3. **C:** Fluoroscopic images of low atrial septum lead position and the PWD during sinus rhythm and low RASp are shown. The vector of the paced P wave is different from that of the sinus P wave, without symmetry in the inferior limb leads and no shortening of PWD, thereby failing to meet the BBp ECG criteria. BBp = Bachmann bundle pacing; ECG = electrocardiogram; LAO = left anterior oblique; PWD = P-wave duration; RAO = right anterior oblique; RASp = right atrial septal pacing.

Atrial pacing leads were successfully implanted in the atrial septum in all cases using the Medtronic C315S5 delivery catheter and the SelectSecure pacing lead. Lead implantation just below the SVC–RA junction, where an abrupt change in atrial electrogram amplitude was observed, was achieved in 40 patients (80.0%), 39 of whom met the BBp ECG criteria. Among the 10 patients in whom atrial lead implantation just below the SVC–RA junction was not achieved, 8 exhibited atrial capture thresholds of >2.0 V before the lead was fixed. In these patients, mapping was extended downward from the SVC–RA junction, and the lead was fixed at a site where the capture threshold was ≤2.0 V. Based on fluoroscopy, 6 leads were positioned in the high atrial septum and 2 in the lower atrial septum. Of the 6 patients with leads implanted in the high atrial septum, 4 met the BBp ECG criteria. Neither patient with leads implanted in the lower atrial septum met the BBp ECG criteria. Furthermore, among the 10 patients in whom atrial lead implantation just below the SVC–RA junction was not achieved, the remaining 2 exhibited no distinct abrupt increase in atrial electrogram amplitude because of diffuse low atrial septal voltage, and the leads were implanted in the atrial septum according to the atrial capture threshold. 1 lead was implanted in the high atrial septum and met the BBp ECG criteria, whereas the other was implanted in the lower atrial septum and did not meet the criteria (Figures 2 and 3).

The atrial mapping time, defined as the duration from insertion of the atrial delivery catheter to lead fixing at the final implantation site, was a median of 134.5 seconds (IQR 71.5–205.5 seconds). The median number of screw deployments was 1 (IQR 1–1), and the mean was 1.28 ± 0.63 (Table 2). The mean unipolar atrial electrogram amplitude before lead fixation was 1.81 ± 0.77 mV. Immediately after fixing the lead, the mean bipolar impedance and capture threshold were 691.1 ± 156.7 Ω and 1.86 ± 1.00 V at 0.4 ms, respectively. Among the 50 patients, 25 (50.0%) showed an initial capture threshold >1.5 V at 0.4 ms immediately after lead fixation; however, in all cases, the threshold improved to ≤1.5 V at 0.4 ms by the end of the procedure. At the end of the procedure, the mean atrial electrogram amplitude, impedance, and capture threshold were 1.95 ± 1.02 mV, 670.7 ± 166.4 Ω, and 0.833 ± 0.315 V at 0.4 ms, respectively. Furthermore, retrospective analysis of electrograms recorded in the EP recording system demonstrated that the mean lead unipolar amplitude averaged over 5 beats was 0.62 ± 0.29 mV on the SVC side and 2.06 ± 0.64 mV on the RA side. The mean delta amplitude across the site demonstrating an abrupt change in electrogram amplitude was 1.45 ± 0.62 mV.

**Table 2:** Procedural characteristics

Variable	
Atrial mapping time, s	134.5 (71.5–205.5)
Number of screw deployments, median / mean	1 (1–1) / 1.28 ± 0.63
Atrial pacing subtypes	
BBp	44 (88.0)
High RASp	3 (6.0)
Low RASp	3 (6.0)
P-wave duration, ms	
SR	143.0 ± 20.2
BBp	118.8 ± 15.4
High RASp	142.7 ± 38.0
Low RASp	141.3 ± 18.8
P-wave amplitude in lead II, mV	
SR	0.090 ± 0.041
BBp	0.137 ± 0.051
High RASp	0.056 ± 0.019
Low RASp	0.010 ± 0.014
Atrial lead parameters at implant	
Sensing amplitude, mV	1.95 ± 1.02
Capture threshold, V at 0.4 ms	0.834 ± 0.315
Pacing impedance, Ω	607.7 ± 166.4

Values are given as numbers (%), means ± standard deviations, or medians (interquartile ranges).

BBp = Bachmann bundle pacing; RASp = right atrial septal pacing; SR = sinus rhythm.

Patients with an initial threshold of >1.5 V (initial high-threshold [iHT] group group) exhibited significantly higher immediate postfixation thresholds and impedances than those with ≤1.5 V (initial low-threshold [iLT] group) (2.59 ± 0.89 V at 0.4 ms vs 1.13 ± 0.34 V at 0.4 ms, *P* < .001; 745.5 ± 188.9 Ω vs 636.8 ± 86.7 Ω, *P* = .014). However, no significant differences were observed between the 2 groups in final capture threshold, impedance, and atrial electrogram amplitude at the end of the procedure (0.84 ± 0.32 V at 0.4 ms vs 0.825 ± 0.31 V at 0.4 ms, *P* = .870; 674.9 ± 181.9 Ω vs 666.5 ± 149.3 Ω, *P* = .863; 1.81 ± 0.68 mV vs 2.08 ± 1.27 mV, *P* = .365) (Table 3). Furthermore, in the iHT group (n = 25), the median recovery time until the capture threshold decreased to ≤1.5 V at 0.4 ms was 367 seconds (IQR 188.5–729 seconds) (Table 3).

**Table 3:** Comparison of lead parameters between the iHT and iLT groups

Variable	iHT (n = 25)	iLT (n = 25)	*P* value
Before lead fixation			
Atrial sensing amplitude, mV	1.8 ± 0.70	1.8 ± 0.83	.915
After lead fixation			
Initial capture threshold, V at 0.4 ms	2.59 ± 0.89	1.13 ± 0.34	<.001
Initial pacing impedance, Ω	745.5 ± 188.9	636.8 ± 86.7	.014
Final capture thresholds, V at 0.4 ms	0.84 ± 0.32	0.83 ± 0.31	.870
Final pacing impedance, Ω	674.9 ± 181.9	666.5 ± 149.3	.863
Final sensing amplitude, mV	1.8 ± 0.70	2.1 ± 1.3	.365
Recovery time, s	367 (188.5–729)	—	

Values are given as means ± standard deviations or medians (interquartile ranges).

iHT = initial high threshold; iLT = initial low threshold.

**Figure 4:**
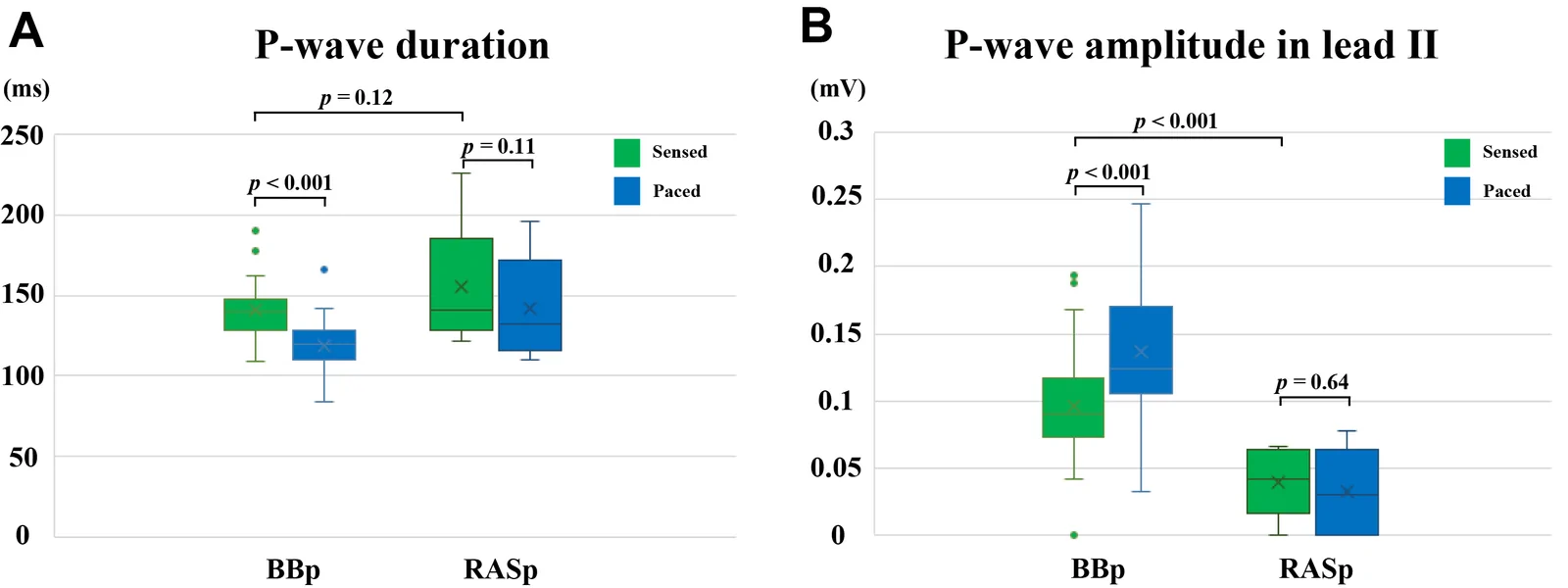
ECG characteristics of BBp and RASp. RASp is defined as atrial septal pacing that does not meet BBp criteria. **A:** Comparison of sensed and paced P-wave duration. **B:** Comparison of sensed and paced P-wave amplitude in lead II. BBp = Bachmann bundle pacing; ECG = electrocardiogram; RASp = right atrial septal pacing.

In patients with successful ECG-defined BBp, the intrinsic PWD and amplitude in lead II were 141.3 ± 16.4 ms and 0.097 ± 0.038 mV, respectively, whereas the paced PWD and amplitude were 118.8 ± 15.4 ms and 0.137 ± 0.051 mV, respectively. The paced PWD was significantly shorter, and the amplitude was significantly higher than intrinsic P waves (*P* < .001 in both). Conversely, among patients with atrial septal pacing who did not meet the BBp ECG criteria, the intrinsic PWD and amplitude were 155.3 ± 35.4 ms and 0.038 ± 0.024 mV, respectively, whereas the paced PWD was 142.0 ± 30.0 ms, showing a nonsignificant trend toward shortening (*P* = .11), and the paced P-wave amplitude (0.033 ± 0.029 mV) was comparable with the intrinsic amplitude (*P* = .64). Regarding baseline P-wave characteristics, no significant difference in intrinsic PWD was observed between patients with successful BBp and those with other RASp (*P* = .12); however, the intrinsic P-wave amplitude in lead II was significantly higher in the BBp group (*P* < .001) (Figure 4).

### Features of local atrial potentials

At the final position, the bipolar electrogram recorded after lead fixation exhibited biphasic or multiphasic potentials in patients who met the BBp ECG criteria. The complex potentials manifested variations, including relatively sharp potentials, low-amplitude high-frequency components, or discrete double potentials, and thus were not uniform in appearance.

### Follow-up, clinical outcomes, and complications

During follow-up at 1 week after implantation (in-hospital) and at 1 and 6 months after discharge, the amplitude, impedance, and capture threshold remained stable. No lead revision because of pacing lead dislodgement, significant increase in atrial capture threshold, or device-related infection was observed. Echocardiography performed during the acute postoperative phase, within 1 week after implantation, revealed no pericardial effusion (Table 4).

**Table 4:** Clinical outcomes and complications

Variable	
All-cause death	0 (0.0)
Heart failure hospitalization	1 (2.0)
New AF onset	5 (10.0)
AF lasting >7 d	0 (0.0)
At the 1-wk follow-up	
Atrial sensing amplitude, mV	2.4 ± 1.4
Capture threshold, V at 0.4 ms	0.69 ± 0.35
Pacing impedance, Ω	574.2 ± 92.6
At the 1-mo follow-up	
Atrial sensing amplitude, mV	2.4 ± 1.3
Capture threshold, V at 0.4 ms	0.75 ± 0.40
Pacing impedance, Ω	555.9 ± 95.4
At the 6-mo follow-up	
Atrial sensing amplitude, mV	2.3 ± 1.3
Capture threshold, V at 0.4 ms	0.91 ± 0.42
Pacing impedance, Ω	541.1 ± 88.0
Complications	
Lead dislodgement	0 (0.0)
Lead revision	0 (0.0)
Pericardial effusion	0 (0.0)
Device-related infection	0 (0.0)

Values are given as numbers (%) and means ± standard deviations.

AF = atrial fibrillation.

### Evaluation of atrial lead position using computed tomography

Among the 50 patients, 15, including 13, were classified under successful ECG-defined BBp and underwent computed tomography (CT) for various reasons during follow-up. Although the atrial lead tips were confirmed to be positioned on the atrial septum in all cases, because CT imaging was not performed with contrast enhancement or ECG gating, detailed anatomic assessment and precise classification of their relationship to the BB area were difficult, beyond confirming that the lead tips were not directed toward the ascending aorta or the RA free wall.

## Discussion

In this study, we demonstrate that atrial septal lead implantation using a lumenless pacing lead delivered through the delivery catheter is safe and that atrial electrogram amplitude–guided identification of the SVC–RA junction allows reproducible placement of the lead at the high atrial septum, fulfilling the BBp ECG criteria. Furthermore, even in cases with high pacing thresholds in the BB area, the use of the C315S5 delivery catheter enabled sequential mapping from the SVC–RA junction to the lower atrial septum, achieving successful atrial septal pacing in all patients.

### Atrial electrogram amplitude–guided approach for BB area mapping

Anatomically, the BB lies just below the SVC–RA junction and serves as the interatrial conduction pathway. As Lustgarten et al^10^ reported in a novel BBp implantation method, techniques for mapping this site include referencing biphasic or multiphasic BB potentials and using anatomic information obtained by injecting a contrast agent using the delivery catheter.

In the present study, an atrial electrogram amplitude–guided approach was used during sinus rhythm to identify the SVC–RA junction. During the procedure, the SVC–RA junction was identified based on unipolar electrogram amplitudes measured using the PSA. The most notable difference from previous techniques is that this approach can be performed using a setup similar to that used in conventional atrial lead implantation. Therefore, in institutions where the use of an EP recording system during device implantation is impractical, or where manpower is insufficient for detailed intraprocedural assessment of paced P-wave morphology using 12-lead ECG, this method may offer advantages in terms of reproducibility. However, if an EP recording system is available for real-time intraprocedural electrogram analysis and detailed paced P-wave evaluation can be performed, combining these modalities would likely further improve the success rate of BBp implantation. Thus, our approach is not intended to discourage the use of the EP recording system or pace mapping, but rather to provide a practical alternative when such resources are limited.

Atrial capture was confirmed at 2 V output at this site before lead fixation; however, the exact threshold and paced P-wave morphology were not assessed. Nevertheless, among the 40 patients in whom the lead was successfully positioned just below the SVC–RA junction, only 1 required remapping, whereas most patients demonstrated favorable pacing thresholds immediately after screw fixation. Furthermore, postoperative evaluation demonstrated that 97.5% of patients with the lead implanted at this site met the BBp ECG criteria, suggesting that this amplitude-guided approach is simple and highly reproducible for ECG-defined BBp implantation. Furthermore, two-thirds of patients who required mapping downward because of high capture thresholds just below the SVC–RA junction, which led to a conventional high atrial septal site, met the BBp ECG criteria. The reason for meeting the BBp ECG criteria even at sites slightly distant from the SVC–RA junction is likely attributable to the fact that the criteria are based on P-wave morphology on surface ECG rather than absolute cutoff values for PWD or P-wave amplitude and that the BB is a broad muscle bundle with individual variation, allowing for effective capture in the relatively wide area of the high RA septum. Regarding predictors of BBp success, sinus P-wave amplitude in lead II was significantly higher in patients with successful BBp and may therefore serve as a predictor of successful ECG-defined BBp using this implantation strategy. This finding may indicate that, in patients with lower sinus P-wave amplitudes who are presumed to have more advanced atrial myocardial disease, structural or electrical abnormalities may also extend to the BB within the high atrial septum, thereby making successful BBp implantation more challenging.

Although it was difficult to accurately depict the atrial lead-tip position using transthoracic echocardiography, all patients who underwent CT were confirmed to have accurate lead placement at the atrial septum. Therefore, lead implantation in the atrial septum using a delivery catheter with a 3D curve was considered safe and feasible.

### Recovery time after atrial lead fixation

According to a report by Nohno et al,^13^ atrial septal lead implantation using the catheter delivery system tended to exhibit a transiently high pacing threshold in the acute phase and time-dependent improvements in pacing thresholds over time. Consistent with this study, we found that the pacing threshold measured immediately after atrial lead fixation was higher than 1.5 V at 0.4 ms in 25 patients (50.0%) in the iHT group. Immediate postfixation thresholds were significantly higher in the iHT group (>1.5 V) than in the iLT group (≤1.5 V) (2.59 ± 0.89 V and 1.13 ± 0.34 V, respectively, at 0.4 ms; *P* < .001). However, at the end of the procedure, the pacing thresholds had sufficiently improved in both groups (0.84 ± 0.32 V and 0.825 ± 0.31 V at 0.4 ms; *P* = .870), and no significant difference was observed between the groups.

The median recovery time from fixing the lead to an improvement in the pacing threshold below 1.5 V was 367 seconds in the iHT group, with the longest case taking 1451 seconds. The impedance immediately after fixing the lead was significantly higher in the iHT group than in the iLT group, and this finding could demonstrate that high impedance indicated intense contact between the pacing lead and the myocardium, suggesting that more severe myocardial injury caused by lead fixation during the acute phase was associated with greater transient threshold elevation. Therefore, when both the lead impedance and threshold are high immediately after lead fixation, the threshold will likely improve over time. Consequently, it may be deemed acceptable to permit a sufficient recovery time of 5 minutes or longer. Furthermore, the potential for severe myocardial injury during atrial septal lead fixation using the catheter delivery system may not only be attributable to the lead perpendicularly attached against the atrial septum but also to the fact that, unlike conventional retractable leads, there is no limit on the number of screw rotations in the lumenless lead design. It is essential to note that prolonged recovery times can lead to an extended total procedure time. Therefore, it is advisable to exercise caution and avoid excessive rotating, given that the manufacturer’s recommended number of screw rotations is approximately 5.

### Local atrial potentials on the atrial lead

Because multiphasic potentials were frequently recorded in the BB area, an implantation technique that uses these potentials as an EP guide for BBp has been reported.^10^ In a recent study demonstrating the efficacy of BBp in patients with nonobstructive hypertrophic cardiomyopathy complicated by interatrial block and heart failure with preserved ejection fraction, the presence of high-frequency electrograms consistent with BB potentials was also included among the criteria for successful BBp.^14^ However, in our previous study involving atrial strain echocardiography, we demonstrated that BBp meeting ECG criteria, including shortened PWD, achieved favorable electromechanical interatrial synchrony.^15^ Based on this finding, we considered BBp that achieves P-wave narrowing to be clinically meaningful, regardless of the presence of an identifiable BB potential. Therefore, the presence of BB potentials was not included in the success criteria for BBp in the present study.

Nevertheless, in the current study, retrospective analysis of local electrograms also demonstrated that patients with successful ECG-defined BBp exhibited multiphasic potentials at the final lead position, supporting the usefulness of this BB potential–guided implantation method. However, 1 of the main reasons for case exclusion in this study was poor recording quality of the lead-tip electrograms because of electrical noise. Although the same EP recording system was routinely used for standard EP studies, intraoperative conditions during device implantation differed significantly from those during EP studies, often making noise elimination from lead-tip potentials technically challenging. The BB potential is usually a complex, low-amplitude electrogram within the P wave, making evaluation difficult when noise levels are high.

In contrast, our implantation method, based on identifying an abrupt change in the atrial electrogram amplitude at the lead tip, relies on amplitude measurements obtained from the Medtronic PSA, as in standard pacemaker implantation. Therefore, this amplitude-guided approach may be particularly advantageous in facilities where an EP recording system is not routinely available.

### Safety and feasibility

At the 6-month follow-up, no cases of lead dislodgement or lead revision because of an increase in pacing threshold or any procedure-related complications such as device-related infection, pericardial effusion, chest pain, pneumothorax, or phrenic nerve capture were observed. The RAA, a conventional atrial pacing site, consists of thin free-wall myocardium, and active-fixation lead implantation in the RAA carries a potential risk of complications involving adjacent thoracic structures, such as pericardial effusion.^16^ In contrast, the atrial septum is anatomically stable and is considered to carry a very low risk of injury to surrounding thoracic structures. Consequently, from a perioperative safety standpoint, the atrial septum seems superior to the RAA as a site for atrial pacing lead implantation.

The absence of lead dislodgement in this cohort may be attributable to 2 technical factors. First, the delivery catheter allows the lead to be positioned and fixed perpendicularly into the atrial septum, ensuring secure fixation. Second, as demonstrated in the previous report,^10^ after fixing the lead into the atrial septum, the operator must gently push down the delivery catheter and lead to withdraw the catheter and create adequate lead slack. When lead dislodgement occurred during this maneuver, we ensured stronger contact between the lead and the septal wall during refixing or occasionally increased the number of screw rotations to achieve stable fixation.

1 important consideration during lead implantation in the BB region is the potential risk of inadvertent lead fixation into the SVC, which may be associated with increased pacing thresholds and lead perforation.^17^ In addition to recognizing the SVC–RA junction and intentionally positioning the lead inferior to this junction, confirmation of injury current after lead fixation may represent an effective strategy to avoid lead fixation within the SVC. Therefore, confirmation of injury current after lead fixation is also recommended. In our practice, injury current was assessed during sinus rhythm from the unfiltered electrograms on the PSA. However, because the present study was retrospective, quantitative analysis of the injury current was not feasible. Nevertheless, a representative example demonstrating the appearance of unipolar injury current during lead fixation is provided in the Supplemental Data.

### Study limitations

The BBp success rate reported in this study was based on retrospective analysis, and lead fixation was not performed in cases with low atrial electrogram amplitude at the high atrial septum where a sudden amplitude change could not be identified or in cases with high pacing thresholds. In such cases, incorporating pace mapping and paced P-wave morphology assessment may further improve BBp implantation success rates. In this context, although sinus P-wave amplitude was significantly higher in patients with successful BBp in our study, it remains unclear whether this finding represents a true general predictor of BBp success or is specific to the implantation strategy used in our study. Furthermore, sites that meet the BBp ECG criteria do not necessarily exhibit favorable lead parameters during the procedure. Therefore, further evidence is needed regarding predictors of procedural success and the long-term efficacy of BBp to better define optimal procedural endpoints.

BBp is defined by ECG criteria; however, it remains uncertain whether the pacing lead was anatomically positioned precisely within the BB area. Transthoracic echocardiography was insufficient for clear visualization of the atrial lead tip, and although CT would be ideal for anatomic assessment, it was not routinely performed in this study to avoid unnecessary radiation exposure. Consequently, CT evaluation was available in only 30% of patients for various reasons. In those limited cases, all atrial leads were confirmed to be positioned in the atrial septum; however, contrast-enhanced CT is required to accurately determine lead-tip location. The definition of successful BBp in the present study relied solely on ECG criteria; therefore, it was not possible to anatomically confirm whether the lead was truly positioned in the BB. As a result, the success rate of BBp implantation may have been overestimated. Future studies are warranted to investigate the correlation between BBp ECG criteria and the precise anatomic location of the implanted lead.

Furthermore, owing to the retrospective design of the study, 15 of the 65 patients were excluded because of insufficient electrogram recordings, potentially introducing selection bias. However, these 15 patients were excluded only from the retrospective analysis; the implantation procedures themselves were completed according to the workflow of the present study. Notably, BBp was successfully achieved in 13 of these 15 patients (86.7%). Therefore, the workflow using this technique seems to be highly reproducible and feasible, regardless of the availability or conditions of the EP recording system.

This single-center, retrospective study had a relatively small sample size and a short follow-up period and was conducted by a limited number of operators experienced with the catheter delivery system for conduction system pacing, limiting its ability to assess the safety and feasibility of this procedure. Future multicenter studies with larger patient populations and longer follow-up are warranted to further validate the efficacy and clinical benefits of BBp.

## Conclusion

The implantation technique for ECG-defined BBp, anticipated to be a physiological atrial pacing, is feasible and reproducibly performed using a Medtronic delivery catheter and lumenless pacing lead. Atrial septal pacing was achieved in all patients, even in those in whom lead implantation at the BB area was not possible because of low amplitude or a high capture threshold. Atrial septal pacing theoretically carries an extremely low risk of extracardiac complications such as pericardial effusion, and the incidence of lead dislodgement was low.

## Disclosures

Dr Yoshimoto has received honoraria and consulting fees from Medtronic. All other authors have no conflicts to disclose.

## Footnotes

Appendix Supplementary data associated with this article can be found in the online version at https://doi.org/10.1016/j.hroo.2026.06.003.
